# In Vitro Susceptibility to Miltefosine of *Leishmania infantum* (syn. *L.* *chagasi*) Isolates from Different Geographical Areas in Brazil

**DOI:** 10.3390/microorganisms9061228

**Published:** 2021-06-05

**Authors:** Caroline Ricce Espada, Erica V. de Castro Levatti, Mariana Côrtes Boité, Dorcas Lamounier, Jorge Alvar, Elisa Cupolillo, Carlos Henrique Nery Costa, Joelle Rode, Silvia R. B. Uliana

**Affiliations:** 1 Departamento de Parasitologia, Instituto de Ciências Biomédicas, Universidade de São Paulo, São Paulo 05508-000, Brazil; caroline.respada@gmail.com (C.R.E.); ericavclevatti@gmail.com (E.V.d.C.L.); 2 Laboratório de Pesquisa em Leishmanioses, Instituto Oswaldo Cruz, Fiocruz, Rio de Janeiro 21040-900, Brazil; maricboite@gmail.com (M.C.B.); elisa.cupolillo@gmail.com (E.C.); 3 Universidade Federal do Piauí, Hospital de Doenças Tropicais Natan Portela, Teresina, Piauí 64002-510, Brazil; dorcas.lc@gmail.com (D.L.); chncosta@gmail.com (C.H.N.C.); 4 Drugs for Neglected Diseases initiative (DNDi), 1202 Geneva, Switzerland; jalvar@dndi.org; 5 Drugs for Neglected Diseases initiative (DNDi), Rio de Janeiro 20010-020, Brazil; jrode@dndi.org

**Keywords:** clinical strains, miltefosine, susceptibility, visceral leishmaniasis

## Abstract

Treatment of visceral leishmaniasis in Brazil still relies on meglumine antimoniate, with less than ideal efficacy and safety, making new therapeutic tools an urgent need. The oral drug miltefosine was assayed in a phase II clinical trial in Brazil with cure rates lower than previously demonstrated in India. The present study investigated the susceptibility to miltefosine in 73 Brazilian strains of *Leishmania infantum* from different geographic regions, using intracellular amastigote and promastigote assays. The EC_50_ for miltefosine of 13 of these strains evaluated in intracellular amastigotes varied between 1.41 and 4.57 μM. The EC_50_ of the 73 strains determined in promastigotes varied between 5.89 and 23.7 μM. No correlation between in vitro miltefosine susceptibility and the presence of the miltefosine sensitive locus was detected among the tested strains. The relatively low heterogeneity in miltefosine susceptibility observed for the 73 strains tested in this study suggests the absence of decreased susceptibility to miltefosine in Brazilian *L. infantum* and does not exclude future clinical evaluation of miltefosine for VL treatment in Brazil.

## 1. Introduction

Visceral leishmaniasis (VL) remains a worldwide public health concern, mainly affecting poor populations across Asia, East Africa, South America, and the Mediterranean region. Over 600 million people live in areas at risk of developing the disease [1]. Overall annual incidence is estimated to be from 50,000 to 90,000 cases, with ten countries concentrating more than 95% of the reported cases: Bangladesh, Brazil, China, Ethiopia, India, Kenya, Nepal, Somalia, South Sudan, and Sudan [1]. In Latin America, VL is a zoonosis caused by *Leishmania infantum* (syn. *L. chagasi*) with dog as the main reservoir of infection and *Lutzomyia longipalpis* the principal vector. It is endemic in 13 countries with an average of 3470 new reported cases every year. In 2019, Brazil reported 2529 new cases, accounting for 97% of cases in Latin America, with around 35% of those in children under 10 years of age [2], and a lethality of 9% [2,3], one of the highest in the world. Another concern is the increasing incidence of HIV/VL coinfection, rising from 0.7% of reported VL cases in Brazil in 2001 to 11.1% in 2019 [2,4].

First line treatment for VL in Brazil is meglumine antimoniate (MA) at a dose of 20 mg intravenous Sb5^+^/kg/day for 20 days. In 2013, the Brazilian Ministry of Health recommended liposomal amphotericin B (LAMB) as the second line treatment, widening its indications. It is currently recommended for patients aged <1 year and >50 years, during pregnancy, with severe illness based on severity score, renal, hepatic, or cardiac damage, HIV-coinfection or other conditions leading to immunodeficiency, therapeutic failure to MA, or other contraindications to MA use [5].

Between 2011 and 2014, a large multicenter clinical trial sponsored by the Brazilian Ministry of Health was conducted to assess the safety and efficacy of treatments recommended for VL in Brazil (MA, amphotericin B deoxycholate, LAMB) and to test a combination of LAMB (10 mg/kg single dose) plus MA for 10 days. Although no statistically significant differences in cure rates were observed, a better safety profile for 3 mg/kg/day LAMB for 7 days, compared to standard MA treatment, was demonstrated [6]. These results are guiding treatment policy change, recommending LAMB monotherapy as the first line treatment option for VL in Brazil. Nonetheless, while LAMB cure rates around 95% have been reported in clinical trials conducted in other countries [7,8,9,10,11,12,13,14], 87.2% cure rates were observed for LAMB in Brazil, suggesting that there is room to improve drug efficacy. This could be achieved through exploring alternative drug combinations with miltefosine, the only current effective oral agent for VL.

However, a clinical trial to assess the efficacy and safety of miltefosine in patients with VL in Brazil conducted in 2005 in Teresina (Piauí) and Montes Claros (Minas Gerais) [15] showed an unsatisfactory overall efficacy at six months of around 60%. A cure rate of 43% was observed in 14 patients from Montes Claros treated with 2.5 mg/kg/day for 28 days. Even with an extension of treatment to 42 days in Teresina, only a 68% final cure rate was reached in these patients. Although differences were not statistically significant, treatment failure was observed in 52.2% of pediatric patients versus 26.3% in adults [16].

The lower cure rate observed in Brazil compared to India could be due to the relatively low dose given to adults (maximum daily dose of 100 mg, while allometric dosing [17,18,19,20] would have required higher daily doses) and the comparatively lower blood levels of miltefosine in children, in line with previous published pharmacokinetic data from India, Nepal, and Africa. On the other hand, previous investigations on the variability of Brazilian *Leishmania braziliensis* strains to miltefosine identified up to 15-fold differences between the susceptibility of strains that had not been exposed to the drug and were obtained from patients prior to treatment [21]. Therefore, reduced susceptibility of Brazilian *L. infantum* strains to the drug could also explain the lower cure rate reported.

A deletion at chromosome 31 detected in several *L. infantum* strains was recently described and associated with an increased risk of miltefosine treatment failure in VL [16]. This locus was named the miltefosine sensitive locus (MSL) and was studied in a sample set of 26 pre-treatment isolates from Montes Claros/Teresina patients who participated in the clinical trial mentioned above. Infection by deleted isolates (absence of MSL) was associated with relapse after miltefosine treatment [15].

In this context, this collaborative project involving the Natan Portella Institute for Tropical Diseases, Federal University of Piauí, Teresina, PI; the Leishmaniasis Laboratory at the Biomedical Sciences Institute of São Paulo University (ICB-USP), São Paulo, SP; the Laboratory of Research on Leishmaniasis-Oswaldo Cruz Institute (IOC-Fiocruz), Rio de Janeiro, RJ; and Drugs for Neglected Diseases *initiative* (DND*i*) aimed to evaluate a large set of Brazilian *L. infantum* strains obtained from humans and dogs presenting VL from different endemic areas in Brazil. The strains were characterized for in vitro susceptibility to miltefosine and genotyped for the presence/absence of MSL.

## 2. Materials and Methods

### 2.1. Ethics Statement

The project was approved by the institutional ethics committees of all the participating institutions: Oswaldo Cruz Institute, Fiocruz (CAAE 76599317.5.0000.5248); Biomedical Science Institute of São Paulo University (CAAE 76599317.5.3002.5467); and Federal University of Piauí (CAAE 76599317.5.3001.5214). Animals used in this study to obtain bone marrow-derived macrophages were treated in accordance with the regulations of the Brazilian Society of Science in Laboratory Animals (SBCAL) and the National Council for Animal Experiment Control (CONSEA), ICB–USP, having obtained the necessary approvals from the institutional ethical committee for animal use.

### 2.2. Chemical Compound

Miltefosine (hexadecylphosphocholine) and amphotericin B were purchased from Sigma-Aldrich (M5571, Lots SLBP4444V and SLBW8818).

### 2.3. Leishmania infantum (syn L. chagasi) Strains

**Figure 1:**
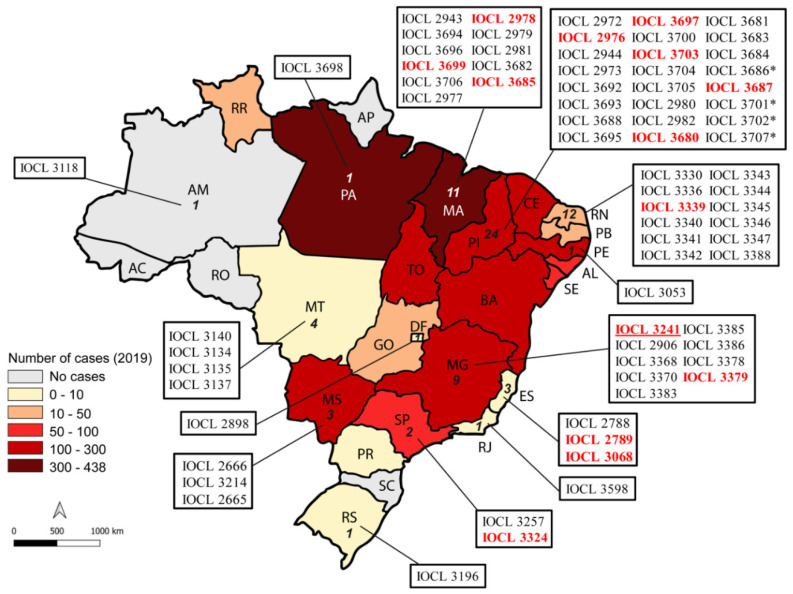
Geographical distribution of *L. infantum* clinical strains tested in this study. The map was colored according to the number of cases registered in 2019 as reported in Brasil [22]. Numbers inside each Brazilian state represent the number of strains that originated there. The reference strain NLC is represented in red and underlined. (*) Strains were allocated according to the geographic region in which the parasite was isolated, when the state in which the infection occurred was not confirmed. Strains used in the initial amastigote assays are highlighted in red.

Seventy-three strains isolated between 2002 and 2015 from humans (*n* = 60) and dogs (*n* = 13) were included in this study. In addition, one *L. infantum* (NLC/IOCL3241) and one *L. donovani* strain were used as internal controls. All *L. infantum* strains employed in this study are deposited in the *Leishmania* Collection at the Oswaldo Cruz Foundation (CLIOC), and were shipped to the ICB-USP Leishmaniasis Laboratory. Isolates were chosen to represent different endemic areas of Brazil (Figure 1), and included six isolates from patients who were enrolled in the clinical trial to assess the efficacy and safety of miltefosine in Teresina [15]. Information provided by the institutions that conducted parasite sampling included the absence of previous treatment before sampling. The activities performed with the *Leishmania* strains were duly registered in SisGen (*Sistema Nacional de Gestão do Patrimonio Genético*) under the identifier AE5FEC8, as determined by article 20 of Decree No. 8.772, in accordance with the Brazilian Biodiversity Law (Law No. 13.123/2015).

### 2.4. Parasite Culture

*L. infantum* promastigotes were grown in sterile M199 medium (Sigma-Aldrich, St. Louis, MO, USA), prepared by diluting the powder in distilled water. Medium was supplemented with 10% heat-inactivated fetal calf serum, 0.25% hemin, 100 μg/mL penicillin/streptomycin, and 2% sterile human urine. Cultures were maintained in an incubator at 25 °C and weekly passages were performed.

### 2.5. Intracellular Amastigote Assay

To determine drug activity in intracellular *L. infantum* amastigotes, bone marrow-derived macrophages (BMDM) from BALB/c mice were obtained using the method previously described [23]. Briefly, 3 × 10^5^ BMDM were left to adhere to round glass coverslips in 24-well culture plates. After incubation in a 5% CO_2_ atmosphere for 24 h at 37 °C, wells were washed with PBS to remove non-adhered cells. Macrophages were then exposed to stationary-phase promastigotes of *L. infantum* at a multiplicity of infection (MOI) of 15 parasites per macrophage for 4 h at 37 °C in a 5% CO_2_ atmosphere.

Non-internalized parasites were removed by successive washings with PBS, and infected macrophages were incubated for 72 h at 37 °C at 5% CO_2_ in RPMI medium with increasing miltefosine concentrations (0.5 to 40 μM). Coverslips were washed with PBS, stained with the Instant Prov kit (Newprov, Pinhais, PR, Brazil) and examined by optical microscopy (Nikon Eclipse E200; Nikon Corporation, Tokyo, Japan). The percentage of infected macrophages, number of amastigotes per infected macrophage, and infectivity index (percentage of infected macrophages multiplied by the average number of amastigotes per macrophage) were determined by counting 100 cells in each triplicate slide. The EC_50_ was determined using the values of the infectivity index through the nonlinear, dose response, sigmoidal model using the GraphPad Prism 6 program. At least two independent experiments were performed in triplicate for each miltefosine concentration and *L. infantum* NCL/IOCL3241 and *L. donovani* Ld15 strains were used as controls in some experiments.

### 2.6. Promastigote Susceptibility Assay

Drug activity was determined by incubating promastigotes of the *L. infantum* strains in the presence of increasing miltefosine (2.5 to 200 µM) and amphotericin B (6.25 to 400 nM) concentrations. After 24 h incubation, viability was assessed by MTT (3-[4,5-dimethyl-2-thiazolyl]-2,5-diphenyl-2H-tetrazolium bromide) assay as previously described [23]. EC_50_ values were obtained by plotting optical density values measured by absorbance at 595 and 690 nm in nonlinear, dose response, sigmoidal models using the GraphPad Prism 6 program. At least three independent experiments were performed in triplicate and *L. infantum* NCL/IOCL3241 and *L. donovani* Ld15 strains were used as controls.

### 2.7. PCR Detection of the Presence/Absence of Miltefosine Sensitive Locus (MSL)

*Leishmania* strains were characterized as MSL- or MSL+ either by whole genome deep sequence analyses or by qPCR. Both methodologies and results are available at Schwabl, et al. [24]. Briefly, to obtain DNA, *Leishmania* strains were grown in biphasic NNN + Schneider’s medium until the end of the log phase, and genomic DNA was extracted using a commercial kit following the manufacturer’s instructions (Qiagen, Valencia, CA, USA). qPCR amplification of the MSL on chromosome 31 or whole genome sequencing was accomplished according to the strategies published [24].

### 2.8. Statistical Analysis

All statistical analyses were performed using Graph Pad Prism 6.0. Correlation between infectivity and EC_50_ of intracellular amastigotes was tested using Spearman’s correlation test. For the analysis of correlation between the presence/absence of MSL locus and EC_50_ to miltefosine, Mann Whitney test was used. Analysis of possible correlations between susceptibility and year of isolation were done using two-tailed unpaired *t*-test with Welch’s. Statistical significance was considered in all these cases for *p*-value under 0.05 (*p* < 0.05).

## 3. Results

**Figure 2:**
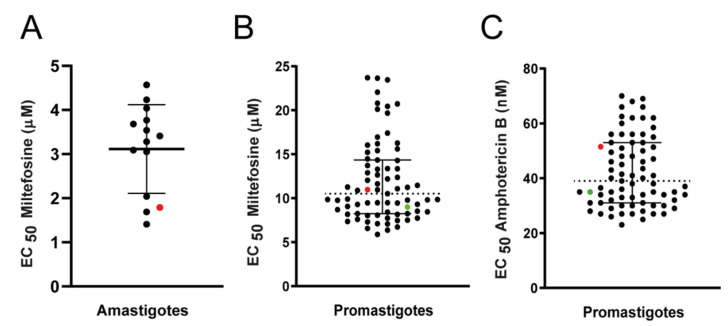
Susceptibility of *L. infantum* strains to miltefosine. Black dots represent the EC_50_ determined for each clinical strain. The red and green dots represent the EC_50_ for *L. infantum* NLC/IOCL3241 (reference strain) and *L. donovani* LD-15, respectively, used as internal controls. Graphics show median EC_50_ (dotted line) and 25% and 75% percentiles (solid lines). (**A**) Susceptibility of *L. infantum* intracellular amastigotes to miltefosine, calculated from at least two independent experiments, each performed in triplicate. (**B**) Susceptibility of *L. infantum* promastigotes to miltefosine. The results shown are the mean of at least three independent experiments, each performed in triplicate. (**C**) Susceptibility of *L. infantum* promastigotes to amphotericin B; mean of two experiments performed in triplicate.

The susceptibility to miltefosine was determined in 13 strains isolated before treatment from 10 humans and 3 dogs, from 5 different Brazilian states and in a control strain (NLC), that was included in these experiments as a reference. The EC_50_ determined in intracellular amastigotes varied from 1.41 to 4.57 μM between the strains (Figure 2A and Table 1). The EC_50_ obtained for the *L. infantum* reference strain NLC to miltefosine was 1.79 ± 0.42 μM (Table 1). Infectivity was also analyzed (Table 1), and correlation tests between EC_50_ of intracellular amastigotes and the percentage of infection revealed that these two variables were not correlated (Spearman’s correlation test: R = −0.2992 and *p* = 0.2965).

In previous studies that evaluated the susceptibility of *Leishmania* strains to miltefosine, we and others observed that, for this drug, there was a direct correlation between susceptibility in amastigotes and promastigotes [21,25]. As promastigote assays are more amenable for larger samples, sensitivity to miltefosine was determined for promastigotes in a total of 73 strains of *L. infantum*, including the 13 strains tested in intracellular amastigotes.

Sensitivity to miltefosine was determined for promastigotes in a total of 73 strains of *L. infantum,* sampled before treatment from 60 humans and 13 dogs. A reference strain was again used as an internal control for all experiments (strain NLC). The *L. donovani* strain LD-15 was also employed as an internal control (Figure 2B, Table 1). The EC_50_ for miltefosine in promastigotes varied from 5.89 to 23.7 μM (Figure 2B, Table 1). The EC_50_ for miltefosine in the *L. infantum* reference strain NLC and the *L. donovani* strain LD-15 were 10.98 ± 1.95 and 9.00 ± 0.80 µM respectively. Calculation of the activity indexes (ratio between the EC_50_ of the clinical strains and EC_50_ of the reference strain) revealed that the highest EC_50_ observed among the clinical strains was 2.16-fold higher than the EC_50_ of the NLC reference strain.

Sensitivity to amphotericin B was also determined in these strains, and a 2.8-fold variation between the lowest (25 nM) and the highest (70 nM) EC_50_ values was observed (Figure 2C, Table 1). The EC_50_ of the *L. infantum* reference strain NLC and of the *L. donovani* LD-15 strain to amphotericin were 51.00 ± 5.00 and 35.00 ± 0.50 nM, respectively.

**Figure 3:**
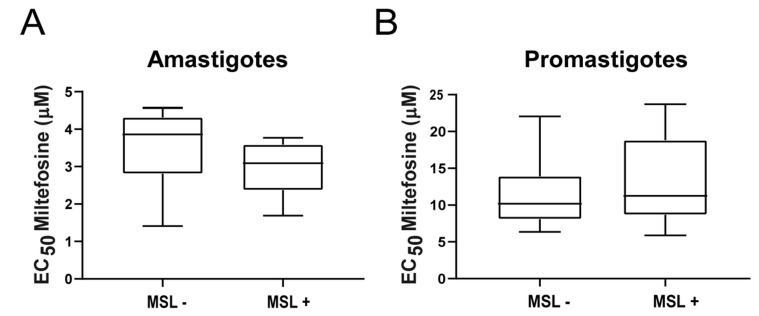
Susceptibility to miltefosine in *L. infantum* strains presenting (MSL+) or not (MSL-) the MSL locus. EC_50_ was determined in amastigotes (**A**) or promastigotes (**B**) and tested for correlation with the genotypic analysis of MSL locus. The central line in the box-plot represents the median values of each analyzed group.

Forty-nine strains (out of 73; 67%) were genotyped for the presence or absence of MSL, recently described and associated with the treatment outcome in Brazilian patients and *L. infantum* in vitro susceptibility to miltefosine [15,16]. Twenty strains (2 from dogs and 18 from humans) were positive (presented) for MSL and 29 (7 from dogs and 22 from humans) were deleted for the locus. No correlations were observed between the EC_50_ for miltefosine and the absence (MSL-) or presence (MSL+) of the locus in amastigotes (*p* = 0.2468; Median for MSL- parasites = 3.86 μM, and for MSL+ = 3.09 μM) (Figure 3A) or promastigotes (*p* = 0.2885; median for MSL- parasites = 10.51 μM, and for MSL+ = 11.25 μM) (Figure 3B).

Statistical analysis was also conducted using only the human *L. infantum* strains from Piauí, Maranhão and Minas Gerais (amastigotes: 2MSL-; 5MSL+; promastigotes: 9MSL-, 18MSL+), aiming to mimic the parasite diversity analyzed previously [16], but even in this restricted population, no statistical differences were observed in the median EC_50_ of MSL- and MSL+ parasites (*p* = 0.3810 and *p* = 0.4948, for amastigotes and promastigotes, respectively) (Figure S1).

**Figure 4:**
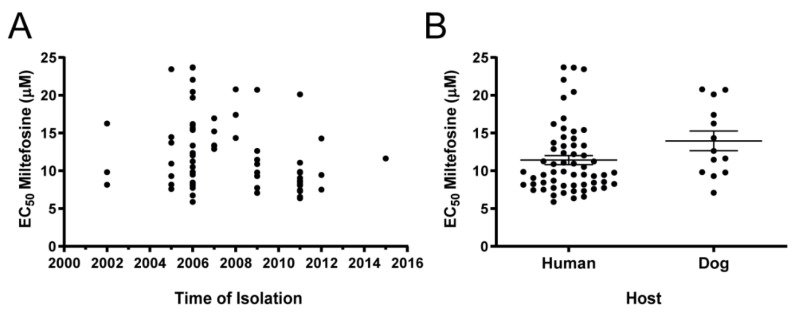
Dispersion analysis of the EC_50_ for miltefosine determined in 73 Brazilian *L. infantum* strains according to (**A**) year of isolation and (**B**) provenance of host. Each black dot represents one clinical strain. In (**B**), the central lines represent mean EC_50_ and SEM of strains. No significant differences between samples originating from humans or dogs were observed (*p* = 0.0950 two-tailed unpaired *t*-test with Welch’s correction).

Analysis of possible correlations between susceptibility and year of isolation detected no significant trends. The same was true for samples isolated from humans and dogs (Figure 4). The analysis of correlation between EC_50_ and geographical origin was not reliable due to low representation for some states (Table 1).

## 4. Discussion

Inadequate efficacy and serious side effects are major and widespread problems of VL treatment. The range of drugs for VL is narrow and fraught with limitations [26]. Miltefosine, though displaying some less than ideal characteristics such as potential teratogenicity, gastrointestinal side effects, and a long plasma half-life of 150–200 h [27], is and will remain for the next few years the only oral drug available for leishmaniasis treatment. Miltefosine also presents a series of advantages compared to the other available treatment options, and care must be taken to avoid the loss of this oral drug. Employed as monotherapy in the Indian subcontinent for several years and in combination therapy with paromomycin as the second line treatment in the framework of the Kala-azar Elimination program [28], miltefosine is not currently in use in Brazil for human visceral leishmaniasis [29,30].

Only one clinical trial to test miltefosine monotherapy for treatment of VL has been conducted in Brazil, with patients enrolled at Montes Claros and Teresina. An overall cure rate of 59% was documented in a sample of 42 treated patients. This study was carried out in two different locations and using two different treatment schedules. In all cases, adults were treated with a 100 mg miltefosine/day total dose, which did not necessarily correspond to the desired dose of 2.5 mg/kg/day [15]. The efficacy in this trial was lower than the 94% cure rate observed in India when miltefosine was implemented [31] and the 90% cure rate observed after a decade of use [29].

Differences in efficacy of the same drug or treatment schedule found in different geographical areas have been demonstrated, but there is no clear understanding of the reasons for this, even given the differences between *L. donovani* and *L.* *infantum* parasite species [28]. This information, and the results obtained in the Brazilian miltefosine trial, raised concerns about the possibility of Brazilian *L. infantum* being intrinsically tolerant to miltefosine. In this context, we gathered efforts to investigate the susceptibility to miltefosine of a panel of clinical strains of *L. infantum* from different geographic regions of Brazil.

The susceptibility to miltefosine of 13 clinical strains was determined for intracellular amastigotes. The EC_50_ varied from 1.41 to 4.57 μM, or 3.2-fold, between the least and the most susceptible isolates. The EC_50_ values in these isolates were strikingly homogenous compared to, for example, Brazilian *L. (V.) braziliensis* clinical isolates, which presented 6- and 15-fold differences in the EC_50_ between the most and the least susceptible parasites, in promastigotes and amastigotes, respectively [21]. This low variability was also reported by Faral-Tello, et al. [32] that evaluated the infectivity and susceptibility of 5 *L. infantum* clinical strains to leishmanicidal drugs including miltefosine and found EC_50_ values that varied from 3.4 to 5.3 μM.

The characterization of susceptibility to miltefosine in a total of 73 strains was done in cultured promastigotes. The promastigote model is less laborious and eliminates the need of animals for macrophage isolation. The susceptibility to amphotericin B was also determined to widen the scope of the study and also to serve as an independent variable in case a heterogeneous behavior toward miltefosine was discovered. The analysis in promastigotes confirmed a low degree of variation (5.89 to 23.7 μM), when compared to the differences found among *L. braziliensis* clinical strains (22.9 to 144.2 μM) [21].

The evaluation of a small percentage of strains in the amastigote stage is a limitation of this study. A correlation between miltefosine susceptibility in promastigotes and amastigotes of *L. braziliensis* [21] and *L. donovani* [25] had already been demonstrated. This correlation was not present among the 13 strains in this study and we hypothesize this could be due to the low variability in the EC50 values determined. However, it would be interesting to widen the spectrum of strains tested as amastigotes in the future. Another aspect that remains to be addressed in future studies is to improve the distribution of strains between the different geographical areas and continuing the evaluation with samples collected more recently. In our sample, some states and samples isolated after 2013 were underrepresented.

The analysis in promastigotes, confirmed a low degree of variation (5.89 to 23.7 μM), when compared to the differences found among *L. braziliensis* clinical strains (22.9 to 144.2 μM) [21].

Recently Carnielli, et al. [16] reported that a deletion of four genes on chromosome 31 found by whole genome sequencing of 26 *L. infantum* strains isolated from cured and relapsed patients from two Brazilian endemic regions was correlated to the treatment outcome. An association between the presence of this locus and a positive response to miltefosine treatment was found, and the genomic region was named miltefosine sensitive locus (MSL) [16]. The susceptibility profile of intracellular amastigotes from strains isolated from patients that relapsed or were cured in the Montes Claros/Teresina clinical trial [15] identified a mean EC_50_ in strains from cured patients of approximately 5 μM whereas for relapsed patients this value was approximately 13 μM. The threshold for predicting treatment outcome was calculated as 8 μM.

In our sample, all the strains presented miltefosine EC_50_ against amastigotes below 5 μM. Due to the high prevalence of MSL deletion in *L. infantum* strains in the Americas [24] more than 60% of the strains studied were genotyped for the presence or absence of the MSL following the previously published protocol [24]. The prevalence of MSL deletion detected in the present study (59%) was in agreement with that found previously (54%) [16]. However, no associations were found between *L. infantum* in vitro susceptibility to miltefosine and the presence/absence of the MSL after testing 49 strains from 11 localities. This lack of association was still observed when the *L. infantum* population analyzed was reduced to geographically mimic the sample previously studied (only from Minas Gerais, Piauí and Maranhão).

Even with the relative homogeneity in the determined miltefosine EC_50_ found in our sample, an important limitation of our study is that the evaluation of in vitro susceptibility does not immediately correlate with the clinical efficacy. Nevertheless, infections of BALB/c mice with the reference strain NLC were successfully treated with 20 mg/kg/day miltefosine for 5 days. When followed up for 4 weeks, these mice showed complete resolution of clinical disease and absence of parasites (M. Galuppo and S. Uliana, personal communication) which supports interpreting the results of the present study as indicating the overall susceptibility to miltefosine of the 73 isolates.

The lack of concordance between data from this and the previous study might reflect differences in methodology, the number of in vitro passages of parasites used, or differences in the populations studied, meaning that there is a need to extend the analysis to a wider sample of strains. However, it is worth considering whether the high failure rates observed in the previous Brazilian clinical trial might have been due to factors other than parasite susceptibility. In the case of *L. donovani* VL, it has been shown that the exposure to miltefosine is lower in children than in adults; indeed, by using an allometric dose in children, efficacy increases from 59% to 90% [19]. Further studies performed to understand the pharmacokinetics and pharmacodynamics of miltefosine in different populations found that the probability of VL relapse was strongly correlated with the time during which the plasmatic concentrations of miltefosine were higher than the EC_90_ values or above 10 × EC_50_ [20]. In African patients, the 2.5 mg/kg/day for 28 days schedule resulted in concentrations above the desired threshold for 51.4 days (CI 30.5–77.1). To analyze the likelihood of failure, the authors employed miltefosine EC_50_ and EC_90_ for *L. donovani* intracellular amastigotes of 4.95 and 25.9 µM, respectively [17]. These values are comparable to the median EC_50_ values for *L. infantum* clinical isolates found in the present study. If pharmacokinetic and pharmacodynamics characteristics in Brazilian patients are comparable to those previously characterized in Nepal and Africa, it is likely that the necessary plasmatic concentrations will be achieved upon use of allometric dosing.

In conclusion, this study indicates that isolates of *L. infantum* obtained over a wide geographical area in Brazil do not appear to be significantly heterogeneous in their susceptibility to miltefosine. Further clinical evaluation of miltefosine for treatment of VL in Brazil is advisable, using an allometric dose in children and associated with pharmacokinetic studies, possibly in combination with other leishmanicidal drugs, in order to evaluate the possibility of incorporating this drug into the therapeutic arsenal for VL treatment in Brazil.

## Figures and Tables

**Table 1:** Descriptive analysis of *Brazilian* isolates and reference strains included in this study.

Identification				Promastigotes				Amastigotes			MSL
				Miltefosine (mM)		Amphotericin B (nM)		Miltefosine (mM)		Infectivity ^g^	
Species ^a^	International Code ^b^	IOCL# ^c^	State ^d^	EC_50_ ± SEM ^e^	AI ^f^	EC_50_ ± SEM ^e^	AI ^f^	EC_50_ ± SEM ^e^	AI ^f^	%	Presence/ Absense ^h^
*L. donovani*	LD-15/MHOM/SD/00	NA	NA	9.00 ± 0.82	0.82	35.0 ± 0.5	NA				NA
*L. infantum*	MHOM/BR/2005/NLC	IOCL3241	MG	10.98 ± 1.95	1.00	51.0 ± 5.0	NA	1.79 ± 0.42	NP	50%	NA
*L. infantum*	MHOM/BR/2009/BLVD	IOCL 3118	AM	7.07 ± 1.63	0.64	58.0 ± 1.0	1.14				MSL-
*L. infantum*	MHOM/BR/2006/NMT-HUB402982MO	IOCL 2898	DF	15.61 ± 0.74	1.42	53.0 ± 6.0	1.04				MSL-
*L. infantum*	MHOM/BR/2005/DRD	IOCL 2788	ES	10.95 ± 1.12	1.0	66.0 ± 4.0	1.29				MSL-
*L. infantum*	MHOM/BR/2005/HRNS-1	IOCL 2789	ES	8.17 ± 1.04	0.74	62.0 ± 3.0	1.22	4.23 ± 0.53	2.36	66%	MSL-
*L. infantum*	MCAN/BR/2008/CP-18	IOCL 3068	ES	17.41 ± 1.77	1.59	70.0 ± 0.0	1.37	3.68 ± 0.05	2.06	39%	MSL-
*L. infantum*	**MHOM/BR/2005/804-NMV**	**IOCL 2943**	MA	9.30 ± 0.40	0.85	48.0 ± 7.0	0.94				NA
*L. infantum*	**MHOM/BR/2006/890**	**IOCL 3694**	MA	5.89 ± 0.11	0.54	50.0 ± 2.0	0.98				MSL+
*L. infantum*	MHOM/BR/2006/6889	IOCL 3696	MA	11.24 ± 1.08	1.02	35.0 ± 4.0	0.69				MSL+
*L. infantum*	MHOM/BR/2006/6905	IOCL 3699	MA	23.65 ± 1.21	2.15	27.0 ± 6.0	0.53	3.05 ± 0.39	1.70	51%	MSL+
*L. infantum*	MHOM/BR/2006/MA07A 4P TMS	IOCL 3706	MA	9.85 ± 1.19	0.9	25.0 ± 3.0	0.49				NA
*L. infantum*	MHOM/BR/2005/841RVSS	IOCL 2977	MA	13.71 ± 1.43	1.25	31.0 ± 0.1	0.61				NA
*L. infantum*	MHOM/BR/2007/1724FRS	IOCL 2978	MA	16.95 ± 1.14	1.54	34.0 ± 9.0	0.67	3.54 ± 1.03	1.98	64%	NA
*L. infantum*	MHOM/BR/2007/1728JLM	IOCL 2979	MA	13.29 ± 0.88	1.21	30.0 ± 8.0	0.59				NA
*L. infantum*	MHOM/BR/2007/1735TAS	IOCL 2981	MA	12.89 ± 1.46	1.17	27.0 ± 5.0	0.53				NA
*L. infantum*	MHOM/BR/2006/P112A 4P NMV	IOCL 3682	MA	19.68 ± 1.39	1.79	20.0 ± 2.0	0.39				MSL+
*L. infantum*	MHOM/BR/2006/MA03-A 4P (GMS)	IOCL 3685	MA	23.70 ± 0.78	2.16	30.0 ± 1.0	0.59	3.77 ± 0.01	2.10	50%	MSL+
*L. infantum*	MHOM/BR/2002/LPC-RPV	IOCL 2906	MG	8.15 ± 1.29	0.74	43.0 ± 16.0	0.84				MSL-
*L. infantum*	MHOM/BR/2011/AD1	IOCL 3368	MG	11.07 ± 1.50	1.01	28.0 ± 2.0	0.55				MSL-
*L. infantum*	MHOM/BR/2011/CR1	IOCL 3370	MG	7.30 ± 1.40	0.66	63.0 ± 1.0	1.24				MSL-
*L. infantum*	MHOM/BR/2012/AD5	IOCL 3383	MG	14.29 ± 0.91	1.3	53.0 ± 5.0	1.04				NA
*L. infantum*	MHOM/BR/2012/AD7	IOCL 3385	MG	7.51 ± 0.91	0.68	39.0 ± 14.0	0.76				NA
*L. infantum*	MHOM/BR/2012/CR2	IOCL 3386	MG	9.45 ± 0.97	0.86	64.0 ± 8.0	1.25				NA
*L. infantum*	MCAN/BR/2010/CA1	IOCL 3378	MG	20.72 ± 1.90	1.89	41.0 ± 11.0	0.8				MSL-
*L. infantum*	MCAN/BR/2010/CA2	IOCL 3379	MG	12.64 ± 1.30	1.15	30.0 ± 2.0	0.59	4.57 ± 1.09	2.55	52%	MSL-
*L. infantum*	MCAN/BR/2002/LVV-137	IOCL 2666	MS	9.82 ± 0.34	0.89	51.0 ± 3.0	1.00				MSL+
*L. infantum*	MCAN/BR/2010/MEG	IOCL 3214	MS	7.10 ± 0.54	0.65	62.0 ± 7.0	1.22				MSL-
*L. infantum*	MCAN/BR/2002/LVV-136	IOCL 2665	MS	16.26 ± 1.36	1.48	26.0 ± 3.0	0.51				MSL+
*L. infantum*	MHOM/BR/2009/202	IOCL 3140	MT	7.73 ± 1.32	0.7	51.0 ± 13.0	1.00				NA
*L. infantum*	MCAN/BR/2009/GRANDÃO I	IOCL 3134	MT	9.31 ± 0.09	0.85	56.0 ± 8.0	1.10				NA
*L. infantum*	MCAN/BR/2009/GRANDÃO II	IOCL 3135	MT	11.46 ± 0.98	1.04	46.0 ± 4.0	0.90				NA
*L. infantum*	MCAN/BR/2009/SOL	IOCL 3137	MT	9.79 ± 0.43	0.89	47.0 ± 3.0	0.92				NA
*L. infantum*	MHOM/BR/2006/791	IOCL 3698	PA	12.37 ± 1.70	1.13	35.0 ± 6.0	0.69				MSL-
*L. infantum*	MHOM/BR/2008/RJS	IOCL 3053	PE	14.35 ± 0.39	1.31	60.0 ± 6.0	1.18				MSL-
*L. infantum*	MHOM/BR/2005/742EMS	IOCL 2972	PI	23.45 ± 1.06	2.14	41.0 ± 5.0	0.80				MSL+
*L. infantum*	MHOM/BR/2006/1406MBS	IOCL 2976	PI	7.73 ± 1.16	0.7	49.0 ± 1.0	0.96	3.27 ± 0.01	1.83	46%	MSL-
*L. infantum*	**MHOM/BR/2005/867-JNS**	**IOCL 2944**	PI	7.59 ± 0.84	0.69	53.0 ± 6.0	1.04				NA
*L. infantum*	**MHOM/BR/2005/792FFS**	**IOCL 2973**	PI	14.47 ± 1.36	1.32	68.0 ± 11.0	1.33				NA
*L. infantum*	**MHOM/BR/2006/891**	**IOCL 3692**	PI	6.73 ± 1.42	0.61	36.0 ± 5.0	0.71				MSL+
*L. infantum*	**MHOM/BR/2006/930**	**IOCL 3693**	PI	12.03 ± 1.08	1.1	62.0 ± 21.0	1.22				MSL+
*L. infantum*	MHOM/BR/2006/P109A 4P JAS	IOCL 3688	PI	8.03 ± 0.42	0.73	35.0 ± 6.0	0.69				MSL+
*L. infantum*	MHOM/BR/2006/867	IOCL 3695	PI	8.45 ± 0.84	0.77	34.0 ± 8.0	0.67				MSL-
*L. infantum*	MHOM/BR/2006/6893	IOCL 3697	PI	8.41 ± 1.21	0.77	37.0 ± 7.0	0.73	3.41 ± 0.75	1.91	69%	MSL+
*L. infantum*	MHOM/BR/2006/6914	IOCL 3700	PI	9.76 ± 1.58	0.89	38.0 ± 3.0	0.75				MSL+
*L. infantum*	MHOM/BR/2006/AAS 3P	IOCL 3703	PI	22.06 ± 1.58	2.01	32.0 ± 8.0	0.63	4.04 ± 0.76	2.26	79%	MSL-
*L. infantum*	MHOM/BR/2006/AAS 4P	IOCL 3704	PI	9.49 ± 1.88	0.86	33.0 ± 9.0	0.65				MSL+
*L. infantum*	MHOM/BR/2006/Pi 11A 4P MBS	IOCL 3705	PI	10.51 ± 0.52	0.96	34.0 ± 12.0	0.67				MSL-
*L. infantum*	MHOM/BR/2007/1732EPG	IOCL 2980	PI	15.22 ± 0.68	1.39	32.0 ± 3.0	0.63				NA
*L. infantum*	MHOM/BR/2007/1739MSS	IOCL 2982	PI	13.37 ± 1.70	1.22	27.0 ± 0.0	0.53				NA
*L. infantum*	MHOM/BR/2006/6909	IOCL 3680	PI	15.41 ± 0.80	1.4	29.0 ± 0.5	0.57	1.69 ± 0.56	0.94	36%	MSL+
*L. infantum*	MHOM/BR/2006/6912	IOCL 3681	PI	13.34 ± 1.13	1.21	26.0 ± 4.0	0.51				MSL+
*L. infantum*	MHOM/BR/2006/JSMG 3P	IOCL 3683	PI	11.26 ± 2.07	1.03	30.0 ± 1.0	0.59				MSL+
*L. infantum*	MHOM/BR/2006/ARS 3P	IOCL 3684	PI	12.20 ± 1.37	1.11	27.0 ± 5.0	0.53				MSL-
*L. infantum*	MHOM/BR/2006/FFS 3P PI-05	IOCL 3687	PI	20.45 ± 1.09	1.86	23.0 ± 1.0	0.45	3.09 ± 0.97	1.73	61%	MSL+
*L. infantum*	MHOM/BR/2006/RUSS-3P	IOCL 3701	PI*	16.04 ± 2.02	1.46	31.0 ± 4.0	0.61				NA
*L. infantum*	MHOM/BR/2006/Pi 04A 4P DHRi	IOCL 3702	PI*	8.25 ± 1.38	0.75	27.0 ± 5.0	0.53				MSL+
*L. infantum*	MHOM/BR/2006/MA01-A 4P	IOCL 3707	PI*	9.46 ± 1.40	0.86	32.0 ± 6.0	0.63				MSL+
*L. infantum*	MHOM/BR/2006/MA04A 4P JNN	IOCL 3686	PI*	16.20 ± 0.78	1.48	31.0 ± 2.0	0.61				MSL-
*L. infantum*	MCAN/BR/2015/TUBO9	IOCL 3598	RJ	11.63 ± 0.48	1.06	34.0 ± 3.0	0.67				MSL-
*L. infantum*	MHOM/BR/2011/Diag 1367	IOCL 3330	RN	8.24 ± 1.13	0.75	36.0 ± 2.0	0.71				MSL-
*L. infantum*	MHOM/BR/2011/TC 03	IOCL 3336	RN	6.56 ± 0.66	0.6	29.0 ± 0.0	0.57				MSL-
*L. infantum*	MHOM/BR/2011/TC 18	IOCL 3339	RN	6.37 ±0.29	0.58	38.0 ± 2.0	0.75	1.41 ± 0.50	0.79	70%	MSL-
*L. infantum*	MHOM/BR/2011/TC 28	IOCL 3340	RN	8.07 ± 1.24	0.73	56.0 ± 16.0	1.10				MSL-
*L. infantum*	MHOM/BR/2011/TC 50	IOCL 3341	RN	9.06 ±0.60	0.83	55.0 ± 4.0	1.08				MSL-
*L. infantum*	MHOM/BR/2011/TC 65	IOCL 3342	RN	9.75 ± 0.60	0.89	41.0 ± 1.0	0.8				MSL-
*L. infantum*	MHOM/BR/2011/TC 95	IOCL 3343	RN	9.86 ± 0.50	0.9	45.0 ± 6.0	0.88				MSL-
*L. infantum*	MHOM/BR/2011/TC 96	IOCL 3344	RN	7.45 ± 0.62	0.68	56.0 ± 9.0	1.10				NA
*L. infantum*	MHOM/BR/2011/TC 98	IOCL 3345	RN	8.53 ± 0.81	0.78	66.0 ± 6.0	1.29				NA
*L. infantum*	MHOM/BR/2011/TC 105	IOCL 3346	RN	9.91 ± 0.76	0.9	43.0 ± 15.0	0.84				NA
*L. infantum*	MHOM/BR/2011/TC 115	IOCL 3347	RN	7.35 ± 0.69	0.67	40.0 ± 3.0	0.78				NA
*L. infantum*	MHOM/BR/2011/TC 86	IOCL 3388	RN	8.69 ± 0.21	0.79	69.0 ± 9.0	1.35				NA
*L. infantum*	MCAN/BR/2010/LUNA II	IOCL 3196	RS	10.88 ± 0.88	0.99	48.0 ± 2.0	0.94				MSL-
*L. infantum*	MCAN/BR/2011/IMTS-14	IOCL 3257	SP	20.11 ± 1.52	1.83	40.0 ± 5.0	0.78				MSL-
*L. infantum*	MCAN/BR/2008/Lchagasi-UFRJ	IOCL 3324	SP	20.79 ± 0.82	1.89	46.0 ± 4.0	0.90	2.04 ± 0.46	1.14	89%	NA

^a^ Two *Leishmania* species, *L. donovani* and *L. infantum* were used in this study. ^b^ International Code for each isolate/strain used in this work. MHOM are isolates obtained from humans, MCAN are isolates from dogs. The strains from patients who participated in the Miltefosine clinical trial (IOCL 2943, IOCL 2944, IOCL 2973, IOCL 3692, IOCL 3693, IOCL 3694) are identified in bold. ^c^ Codes for *Leishmania* strains deposited in the *Leishmania* Collection at Fundação Oswaldo Cruz (CLIOC). ^d^ Brazilian State of origin of the isolates. AM = Amazonas; DF = Distrito Federal; ES = Espírito Santo; MA = Maranhão; MG = Minas Gerais; MS = Mato Grosso do Sul; MT = Mato Grosso; PA = Pará; PE = Pernambuco; PI = Piauí; RJ = Rio de Janeiro; RN = Rio Grande do Norte; RS = Rio Grande do Sul; SP = São Paulo. PI* Brazilian State of the institution that sent the isolates (State of origin of sample not confirmed). ^e^ EC_50_ ± SEM of miltefosine determined for promastigotes (in three independent experiments) and amastigotes (in two independent experiments) and of amphotericin B for promastigotes (also in two independent experiments). ^f^ Activity index was calculated by dividing the EC_50_ of each clinical isolate by the EC_50_ of the *L. infantum* reference strain NLC. NP = not applicable. ^g^ Mean percentage of infection obtained for each isolate/strain after 72 h of infection and in the absence of drug. ^h^ MSL = miltefosine sensitivity locus. MSL+ indicates the presence of the locus in homozygosis; MSL- absence of the referred locus in homozygosis; NA = not available. Data reported in Schwabl, et al. [24].

## Data Availability

The data presented in this study are available in the article and supplementary material.

## Supplementary Materials

The following are available online at https://www.mdpi.com/article/10.3390/microorganisms9061228/s1. Figure S1: Susceptibility to miltefosine in *L. infantum* strains presenting (MSL+) or not (MSL-) the MSL locus.
