# Supplementary material for: In Vitro Susceptibility to Miltefosine of Leishmania infantum (syn. L. chagasi) Isolates from Different Geographical Areas in Brazil
Source: Microorganisms. 2021 Jun 5;9(6):1228. doi: 10.3390/microorganisms9061228 (PMC8228039; doi:10.3390/microorganisms9061228)
Supplement: Supplementary file 1 [file microorganisms-09-01228-s001.zip › microorganisms-1182473-supplementary.pdf]

Supplementary Materials

In Vitro Susceptibility to Miltefosine of *Leishmania infantum* (syn. *L. chagasi*) Isolates from Different Geographical Areas in Brazil.

Caroline Ricce Espada [1,†], Erica V. de Castro Levatti [1,†], Mariana Côrtes Boité [2], Dorcas Lamounier [3], Jorge Alvar [4], Elisa Cupolillo [2], Carlos Henrique Nery Costa [3], Joelle Rode [5] and Silvia R. B

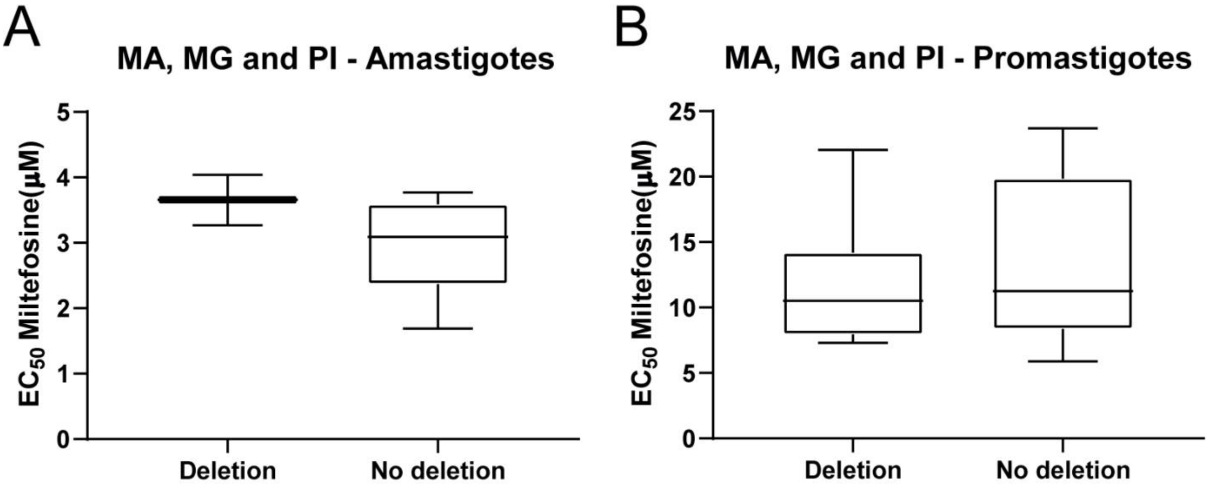

Figure S1. Susceptibility to miltefosine in *L. infantum* strains presenting (MSL+) or not (MSL-) the MSL locus. EC_{50} was determined in amastigotes (A) or promastigotes (B). Distribution analysis performed with strains originating from Piauí, Maranhão and Minas Gerais Brazilian states. The central line in the box-plot represents the median values of each analyzed group.
